# Indication to dynamic and invasive testing in Cushing’s disease according to different neuroradiological findings

**DOI:** 10.1007/s40618-021-01695-1

**Published:** 2021-10-26

**Authors:** E. Ferrante, M. Barbot, A. L. Serban, F. Ceccato, G. Carosi, L. Lizzul, E. Sala, A. Daniele, R. Indirli, M. Cuman, M. Locatelli, R. Manara, M. Arosio, M. Boscaro, G. Mantovani, C. Scaroni

**Affiliations:** 1 Endocrinology Unit, Fondazione IRCCS Ca’ Granda Ospedale Maggiore Policlinico di Milano, Via Francesco Sforza, 35, 20122 Milan, Italy; 2 Endocrinology Unit, Department of Medicine DIMED, University of Padova, Padua, Italy; 3 Department of Experimental Medicine, Sapienza University of Rome, Rome, Italy; 4 Department of Clinical Sciences and Community Health, University of Milan, Milan, Italy; 5 Neurosurgery Department, Fondazione IRCCS Ca’ Granda Ospedale Maggiore Policlinico di Milano, Milan, Italy; 6 Department of Pathophysiology and Transplantation, University of Milan, Milan, Italy; 7 Department of Neurosciences, University of Padua, Padua, Italy

**Keywords:** Cushing’s disease, ACTH-dependent Cushing’s syndrome, Differential diagnosis, Bilateral inferior petrosal sinus sampling

## Abstract

**Purpose:**

Dynamic testing represents the mainstay in the differential diagnosis of ACTH-dependent Cushing’s syndrome. However, in case of undetectable or detectable lesion < 6 mm on MRI, bilateral inferior petrosal sinus sampling (BIPSS) is suggested by current guidelines. Aim of this study was to analyze the performance of CRH, desmopressin and high-dose dexamethasone suppression test (HDDST) in the differential diagnosis of ACTH-dependent Cushing’s syndrome as well as the impact of invasive and noninvasive tests on surgical outcome in patients affected by Cushing’s disease (CD).

**Methods:**

Retrospective analysis on 148 patients with CD and 26 patients with ectopic ACTH syndrome.

**Results:**

Among CD patients, negative MRI/lesion < 6 mm was detected in 97 patients (Group A); 29 had a 6–10 mm lesion (Group B) and 22 a macroadenoma (Group C). A positive response to CRH test, HDSST and desmopressin test was recorded in 89.4%, 91·4% and 70.1% of cases, respectively. Concordant positive response to both CRH/HDDST and CRH/desmopressin tests showed a positive predictive value of 100% for the diagnosis of CD. Among Group A patients with concordant CRH test and HDDST, no difference in surgical outcome was found between patients who performed BIPSS and those who did not (66.6% vs 70.4%, *p* = 0.78).

**Conclusions:**

CRH, desmopressin test and HDDST have high accuracy in the differential diagnosis of ACTH-dependent CS. In patients with microadenoma < 6 mm or non-visible lesion, a concordant positive response to noninvasive tests seems sufficient to diagnose CD, irrespective of MRI finding. In these patients, BIPSS should be reserved to discordant tests.

## Introduction

Cushing’s syndrome (CS) is a rare and potentially fatal condition due to chronic exposure to cortisol. After excluding exogenous glucococorticoid assumption from any route, the diagnosis is based on clinical suspicion and further confirmed with appropriate testing as suggested by Endocrine Society Guidelines [urinary free cortisol (UFC), late night serum/salivary cortisol and 1 mg dexamethasone suppression test] [1]. Once the diagnosis of endogenous hypercortisolism is confirmed, the measurement of morning ACTH levels allows to discriminate ACTH-dependent from ACTH-independent CS that originates from primary adrenal disorders. Among ACTH-dependent CS, the most common form is caused by an ACTH-secreting pituitary tumor, a condition named Cushing’s disease (CD), accounting for about 80% of all cases, whereas the rest is due to an ectopic source (EAS); even though ACTH levels are usually higher in EAS than in CD, there is a significant overlap between these two conditions, thus further diagnostic procedures are needed [1]. Desmopressin (DDAVP) stimulatory test is helpful in suggesting risk of recurrence in the post-neurosurgical follow-up, but it seems to have a limited diagnostic utility in the differential diagnosis of ACTH-dependent CS due to the expression of vasopressin receptors in both CD and EAS [2]. Conversely, high-dose dexamethasone suppression test (HDDST) and corticotropin-releasing hormone (CRH) test have been widely used for this purpose and represent the mainstay in the differential diagnosis of ACTH-dependent CS forms [3–6]. Despite their satisfactory accuracy, there is no consensus on how to interpret their results [7]. Previous studies found that the presence of concordant clear-cut response to both HDDST and CRH test is able to exclude the diagnosis of EAS, irrespective of magnetic resonance imaging (MRI) finding [8, 9]. Even though MRI with intravenous gadolinium administration is certainly useful for individuation of the pituitary tumor, it results in little help in about 30% of cases due to tiny dimensions, localization and characteristics of the ACTH-secreting pituitary adenomas [10]. Conversely, radiological studies may sometimes disclose abnormalities with no functional significance, the so-called “pituitary incidentalomas”, that have been found in about 10% of healthy individuals [11], as in up to 38% of patients with EAS [12]. However, it is noteworthy that the finding of a pituitary incidentalomas larger than 6 mm in patients with EAS is usually very rare [13]. The presence of a microadenoma is therefore not enough for hypercortisolism to be labeled as pituitary-dependent and the role of hormonal tests is crucial for a correct diagnosis. When discordant results to dynamic tests and/or when pituitary MRI shows a lesion < 6 mm, bilateral inferior petrosal sinus sampling (BIPSS) is still recommended as the gold-standard procedure to achieve correct differential diagnosis due to its high sensitivity and specificity [7]. However, even BIPSS is not always fully reliable; false negative results are indeed possible in case of anatomical variations of the venous drainage from the cavernous sinuses to the jugular veins or when BIPSS is performed in a low-normal cortisolemic phase, as might happen in cyclic CS or during treatment with cortisol-lowering medications [14]. Furthermore, BIPSS requires hospitalization, is time- and cost-consuming and in few instances might lead to severe complications [15, 16]. Given the fact that BIPSS is not 100% accurate, has poor reliability to suggest intrapituitary localization/lateralization and has some drawbacks [17], we collected clinical, biochemical and neuroradiological data of a large series of CD patients as well as biochemical and neuroradiological data of a group of EAS patients with the following aims: (i) to describe the responsiveness to dynamic testing (CRH test, DDAVP test and HDDST) and its performance in the differential diagnosis of ACTH-dependent Cushing’s syndrome in possible different scenarios given by MRI finding; (ii) to assess whether the decision of BIPSS execution can affect surgical outcome of patients affected by Cushing’s disease.

## Patients and methods

We performed a retrospective analysis on 148 patients (F/M 113/35, mean age 42.4 ± 14.2 years) affected by CD followed at 2 tertiary care centers in Italy between 2000 and 2017 [Endocrinology Unit, Fondazione IRCCS Ca’ Granda Ospedale Maggiore Policlinico of Milan (62 patients); Endocrinology Unit, Department of Medicine-DIMED, University of Padova (86 patients)].

The diagnosis of hypercortisolism was performed on the basis of typical clinical features in the presence of at least two of the following abnormal tests: high 24-h UFC levels, loss of circadian rhythm in plasma/salivary cortisol and lack of cortisol suppression after 1 mg of dexamethasone overnight [1]. The diagnosis of ACTH-dependent hypercortisolism was confirmed in case of detectable baseline ACTH plasma levels (> 20 ng/L) [18]. Pituitary MRI (magnet strength ranging from 1.5 to 3.0 TESLA over the study period) with gadolinium was performed in all patients and reviewed by experienced neuroradiologists. Differential diagnosis of ACTH-dependent hypercortisolism was established through: (i) CRH test (positive response: ACTH and/or cortisol plasma levels increase by more than 50% and/or 20%, respectively) [12, 18–20]; (ii) high-dose dexamethasone suppression test (HDDST) (positive response: serum cortisol levels reduction to a value of < 50% of the basal level) [19]; (iii) DDAVP test (positive response: increase of both ACTH and cortisol greater than 30% and 20%, respectively) [21, 22].

For CRH and DDAVP tests, all patients were evaluated after an overnight fast; blood samples for ACTH and cortisol measurements were collected − 15, 0, 15, 30, 45, 60, 90 and 120 min after intravenous bolus injection of human CRH 100 µg or DDAVP 10 µg, respectively.

For HDDST, dexamethasone 8 mg was administered orally at 23.00 h and serum cortisol levels were measured between 8.00 and 9.00 a.m. on the next morning.

The decision whether to perform bilateral inferior petrosal sinus sampling (BIPSS) was guided by clinical judgement considering neuroradiological and biochemical findings. After catheter placement, ACTH was measured simultaneously in a blood sample obtained from each petrosal sinus and from a peripheral vein before and 1, 3, 5, and 10 min after the injection of 1 µg/Kg of CRH.

An inferior petrosal sinus to periphery ratio (IPS:P) ≥ 2 at baseline or ≥ 3 after CRH administration was considered as positive response [23]. All patients included in this study underwent transsphenoidal surgery (TSS) performed by neurosurgeons with recognized expertise in the management of pituitary diseases.

The pituitary origin of ACTH secretion was then confirmed by immediate (serum cortisol < 138 nmol/L within 7 days following TSS) and/or sustained biochemical remission [hypoadrenalism (morning serum cortisol < 138 nmol/L or lack of cortisol response to Synacthen stimulation test considering a cut-off of 500 nmol/L) for at least 6 months] after TSS and/or histological examination (defined as positive immunostaining for ACTH on the adenomatous tissue).

Finally, data describing biochemical responses to CRH test, DDAVP test and HDDST and pituitary MRI in a group of 26 patients (14 of which were presented in a previous publication) [9] with histologically confirmed ectopic ACTH syndrome (EAS) were also collected.

### Statistical analysis

Data are shown using mean ± standard deviation for normally distributed continuous variables or median and interquartile range (IQR) for non-Gaussian data and proportion for categorical parameters. Categorical data were analyzed using the χ^2^ test or the Fisher exact test if the expected value was < 5. Continuous parameters with normal distribution were compared using the *t* test and non-Gaussian data using the non-parametric test of Mann Whitney. The relation between two or more variable was assessed through logistic regression in case of binary dependent variable and linear regression in case of continuous dependent variable. Sensitivity (SE), specificity (SP), positive predictive value (PPV) and negative predictive value (NPV) were calculated with 95% confidence intervals (CI) using the exact binomial method. All statistical analyses were performed using SPSS, version 25 (IBM, Cary, NC, USA).

## Results

### Neuroradiological findings

**Fig. 1:**
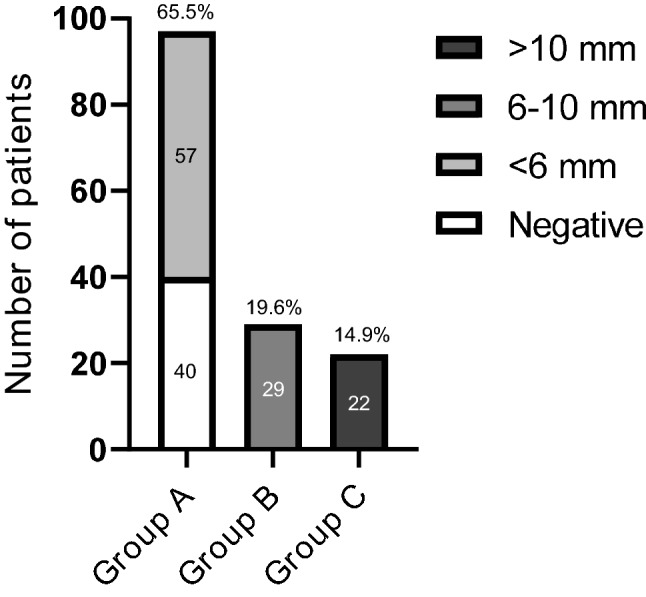
Different groups of patients according to MRI findings

Patients with CD were divided into three groups on the basis of MRI results; group A included 97 patients (65.5%) with negative imaging (*n* = 40, 27% of total) or with a pituitary lesion < 6 mm (*n* = 57 patients, 38.5%); group B those with visible pituitary adenoma sized between 6 and 10 mm (29 subjects, 19.6%), while group C accounted for patients with macroadenoma (22 patients, 14.9%) (Fig. 1).

Among patients with EAS, seven had a microadenoma < 6 mm, while pituitary imaging was negative in 19.

### Biochemical characteristics at baseline

Demographic, basal and dynamic biochemical characteristics and remission rates of three groups of patients affected by CD are summarised in Table 1.

**Table 1:** Demographic, basal and dynamic biochemical characteristics and remission rates of three groups of patients

	Group A (*n* = 97)	Group B (*n* = 29)	Group C (*n* = 22)	*p*
Age (years) (mean ± SD)	42.8 ± 14	37.8 ± 13	46.9 ± 15.3	0.09
Sex (F/M)	74/23	24/5	15/7	0.48
Basal cortisol (nmol/L) (median, IQR)	578 (454.5–692.7)	579 (468–775.3)	543 (486.3–695.7)	0.93
ACTH (pg/ml) (median, IQR)	43 (33–58.6)	57 (34.2–77.5)	90 (54.5–113.5)	< 0.001
Relative UFC (median, IQR)	2.4 (1.6–3.9)	2.5 (1.4–3.1)	2.2 (1.3–5.1)	0.93
Cortisol nadir after HDDST (nmol/L) (median, IQR)	96.5 (64.6–141.7)	95.9 (51.7–178.7)	157 (57.3–230.5)	0.64
Cortisol reduction after HDDST (%) (median, IQR)	81.7 (73.8–88.7)	83.3 (73.6–89.1)	76.9 (57.1–88.9)	0.53
ACTH increase after CRH (%) (median, IQR)	143.3 (59.7–285.7)	115.4 (77.9–299.3)	82 (26.4–190.9)	0.07
Cortisol increase after CRH (%) (median, IQR)	61.8 (31.4–97.7)	60.4 (27.9–76.1)	36.8 (15.6–63.1)	0.07
ACTH increase after DDAVP (%) (median, IQR)	106.8 (24–283.7)	111.2 (27.3–279.4)	67.3 (45.8–261.4)	0.94
Cortisol increase after DDAVP (%) (median, IQR)	38.6 (18.1–76.2)	40.5 (15.3–87.8)	40.4 (24.6–88.0)	0.72
Surgical remission (*n*/tot, %)	68/97 (70.1%)	25/29 (86.2%)	14/22 (63.6%)	0.14

Basal levels of cortisol, ACTH and UFC were evaluated for each group. Because of different assay methods performed during time, we preferred to use relative UFC (UFC/upper normal limit ratio). Patients of Group C showed higher basal ACTH levels compared to patients with negative MRI imaging or microadenomas (Group A + B) [90(54.5–113.5) vs 44.6(33.7–65.6), *p* < 0.001), without difference between Group A and Group B. No difference in basal cortisol and relative UFC levels was found between groups.

Late night salivary cortisol levels were evaluated in 73 patients (47 of Group A, 13 of Group B and C) without any difference between groups.

### Suppression test

Overall, a positive response to HDDST was observed in 91.4% of cases of CD. The rate of responders to HDDST was similar between negative MRI/microadenomas (Group A + B) and macroadenomas (respectively 92.6% vs 83.3%, *p* = 0.18) and no differences were found in cortisol levels and percentage of cortisol reduction after HDDST among the three different groups of patients (Table 1).

Six out of 26 patients affected by EAS were responsive to HDDST (23.1%). HDDST had a 91% SE, 77% SP, 95% PPV and 62% NPV to diagnose Cushing’s disease (Table 2).

**Table 2:** Diagnostic performance of positive response to CRH test, HDDST and their combination for the correct identification of Cushing’s disease

	Whole cohort (*n* = 174)			Negative MRI or adenoma < 6 mm (*n* = 123)		
	CRH	HDDST	CRH + HDDST	CRH	HDDST	CRH + HDDST
Sensitivity	89 (83–93)	91 (86–95)	81 (74–87)	91 (84–95)	92 (85–96)	82 (74–89)
Specificity	96 (92–98)	77 (70–83)	100 (97–100)	96 (90–99)	77 (68–84)	100 (96–100)
PPV	99 (96–100)	95 (91–98)	100 (97–100)	99 (94–100)	94 (87–97)	100 (96–100)
NPV	62 (55–70)	62 (55–70)	51 (43–59)	76 (67–83)	71 (62–79)	62 (52–70)

Values are reported as a percentage and 95% confidence intervals

*CRH* corticotropin-releasing hormone, *HDDST* high-dose dexamethasone suppression test, *PPV* positive predictive value, *NPV* negative predictive value

### Dynamic tests

Overall, CRH test was positive in 89.4% of CD subjects. The response rate was significantly higher in patients with negative MRI/microadenomas (Group A + B) with respect to those with macroadenomas (91.7% vs 75%, *p* = 0.04), without difference between Group A and Group B. Likewise, negative MRI/microadenomas showed a higher response in terms of ACTH [140.5 (71.9–284.9) vs 82 (26.4–190.9) *p* = 0.02] and cortisol percentage increase [61.8 (30.7–92.8) vs 36.8 (15.6–63.1), *p* = 0.03].

As far as DDAVP is concerned, a positive response was recorded in 70.1% of the whole cohort. In this case, unlike CRH test, the response rate was significantly higher in patients with macroadenomas than in those with negative MRI/microadenomas (90% vs 66.3%, *p* = 0.03). However, no differences between negative MRI/microadenomas and macroadenomas in terms of percentage increase of ACTH and cortisol were found.

Concordance of positive responses between CRH test and HDDST was observed in 81.5% of all patients (82.4% in Group A, 88.4% in Group B and 66.6% of Group C) without any difference between groups. In four cases, a negative response to both tests was recorded; all these patients had a macroadenoma with a minimum diameter of 20 mm.

Concordant positive responses to CRH and DDAVP tests were observed in 62.6% of patients (62.9% in Group A, 56.5% in Group B and 68.4% in Group C, *p* = NS between groups). In Group A, the concordance rate between CRH and DDAVP was significantly lower than that observed between CRH test and HDDST (62.9% vs 81.5%, *p* = 0.035). Additionally, six patients (four of Group A, one of Group B and one of Group C) showed a negative response to both tests.

With regards to EAS, one patient had a positive response to CRH test and six patients to HDDST, respectively. Data regarding DDAVP test were available in 22 out of 26 patients: in this subgroup, a false positive response was observed in 11 patients. However, no patient showed a concordant positive response to CRH test and HDDST or to CRH test and DDAVP test. Conversely, two patients responded to both HDDST and DDAVP test. Although it is beyond the aim of this paper, our data confirm previous studies reporting a higher sensitivity of CRH in respect to HDDST and DDAVP test in this setting [24–26].

CRH test showed a SE of 89%, SP of 96%, PPV of 99% and NPV of 62% for the diagnosis of CD (Table 2). The combination of the concordant positive responses to CRH test and HDDST performed better than single tests, reaching a 100% SP and PPV irrespective of pituitary MRI.

Considering only the patients with negative imaging or a pituitary lesion < 6 mm, the SE, SP, PPV and NPV of combined positive responses were 82%, 100%, 100% and 62%, respectively (Table 2). On the other hand, combined negative responses in this subgroup of patients showed a SP and PPV of 100% for the diagnosis of EAS.

Similarly, a positive response to both CRH test and DDAVP test reached a SP and PPV of 100% for the diagnosis of CD (Table 3).

**Table 3:** Diagnostic performance of positive response to DDAVP test or to the combination DDAVP/CRH and DDAVP/HDDST for the correct identification of Cushing’s disease

	Whole cohort (*n* = 170)			Negative MRI or adenoma < 6 mm (*n* = 119)		
	DDAVP	DDAVP + CRH	DDAVP + HDDST	DDAVP	DDAVP + CRH	DDAVP + HDDST
Sensitivity	70 (62–77)	63 (54–70)	66 (57–73)	68 (58–76)	63 (53–72)	63 (53–72)
Specificity	50 (42–58)	100 (97–100)	91 (85–95)	50 (40–60)	100 (96–100)	91 (83–95)
PPV	89 (83–93)	100 (97–100)	98 (93–99)	84 (75–90)	100 (96–100)	96 (90–99)
NPV	22 (16–30)	32 (25–40)	32 (25–41)	29 (21–39)	42 (33–52)	39 (30–49)

Values are reported as a percentage and 95% confidence intervals

*CRH* corticotropin-releasing hormone, *HDDST* high-dose dexamethasone suppression test, *PPV* positive predictive value, *NPV* negative predictive value

### Bilateral inferior petrosal sinus sampling in CD

BIPSS was performed in 29/97 patients of Group A and 1/29 patient of Group B. In particular, 20 of 29 patients of Group A had a negative MRI. In four out of these patients, CRH and HDDST were discordant (two negative results for each test) and BIPSS confirmed a pituitary origin of CS. In the other 16 cases, a positive response to both tests was observed: in 15 cases BIPSS confirmed the diagnosis of CD, while a central/periphery ratio of 2.91 after CRH administration was recorded in one case. The latter patient underwent TSS and CD was then confirmed by immediate and long-term remission of disease. Notably, no patient of Group A presented a negative response to both CRH test and HDDST, while four patients presented a combined negative response to CRH and DDAVP tests.

In the remaining nine patients of Group A, MRI showed a visible microadenoma < 6 mm and BIPSS confirmed the diagnosis of CD both in concordant (*n* = 6) and discordant (*n* = 3) patients.

BIPSS was not consistent with a pituitary origin in a patient of Group B with discordant tests. However, as her pretest probability of having CD was high (she was a young female without any suggestive features of ectopic CS and no lesion at thoracoabdominal computed tomography), also in this case the patient underwent TSS and both short and long-term remission confirmed the diagnosis of CD.

No complications were observed in 29/30 patients after BIPSS. One patient died about 24 h after the procedure because of cardiac rupture. Since autopsy revealed a left ventricular free-wall rupture after asymptomatic acute myocardial infarction and cortisol related myopathy, this event was considered as unlikely related to BIPSS.

### Remission rates after surgery and role of BIPSS in CD patients with inconclusive neuroradiological imaging

Overall, surgical remission was achieved in 107/148 (72.3%) patients. No difference between groups was found, also considering all patients with negative MRI or microadenomas (Group A + B) with respect to those with macroadenomas (Group C) (73.8% vs 63.6%, *p* = 0.31).

**Fig. 2:**
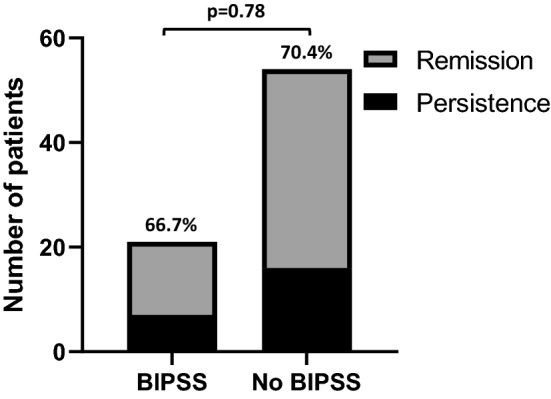
Remission rate in patients of Group A with concordant positive tests

Finally, when considering patients of Group A with concordant positive responses to HDDST and CRH test (*n* = 75), no difference in surgical outcome was found between patients who performed BIPSS and those who did not [respectively, 14/21 (66.6%) vs 38/54 (70.4%), *p* = 0.78] (Fig. 2).

## Discussion

Differential diagnosis of ACTH-dependent CS is challenging and to date a single best approach in the diagnostic work-up of these patients does not exist.

Whereas the usefulness of stimulatory and suppression tests is widely accepted, their role to the light of positive MRI (pituitary adenoma < or > 6 mm) or negative findings is still a matter of debate. In the latter case, although BIPSS still represents the gold-standard procedure for differential diagnosis regardless the results of dynamic tests [7, 18], different clinical approaches and opinions are reported in the literature.

In a recent opinion statement by members of the Italian Society of Endocrinology, Italian Society of Neurosurgery and Italian Society of Neuroradiology that summarizes different strategies adopted in the prescription of BIPSS [27], the authors report two studies in which BIPSS did not show any influence on neurosurgical remission rates. In the first one, Bochicchio and coll. retrospectively analyzed data from 668 patients affected by CD and described that in 98 subjects who underwent BIPSS, surgical failure was similar to patients who did not [28]; however, in this cohort CRH and TRH tests but not HDDST, were performed and selection criteria for BIPSS were not clearly reported. In the second one, Jehle and coll. performed a retrospective analysis of 193 patients with ACTH-dependent CS [29]; also in this case, BIPSS did not affect remission rate after TSS as far as recurrence and long-term remission rates. The procedure was reserved to patients with equivocal scan and/or biochemical tests; however, biochemical evaluation consisted of ACTH and UFC levels, while CRH test was not performed and data about HDDST were lacking in all but six patients.

In a subsequent review about the role of BIPSS in CS, Zampetti et al. [30] suggested that, on the basis of authors’ experience, BIPSS should not be performed in patients with positive response to CRH test (defined as increase > 50% in ACTH and > 30% in cortisol), particularly if a consistent suppression to HDDST is present, independently of MRI findings. This opinion was finally remarked by Losa et al. [14] which pointed out CRH test as the main factor in providing indication to BIPSS.

In this area of controversy, we performed a retrospective analysis on 148 patients with CD and 26 patients with EAS aiming to evaluate the role non-invasive tests in the diagnostic work-up, with secondary focus on the need of BIPSS in CD patients with inconclusive neuroradiological examination. In all 148 patients of our cohort, the diagnosis of CD was confirmed by biochemical remission after TSS, histology and/or > 6 months post-surgical hypoadrenalism.

In agreement with previous data, our results confirm that CRH test and HDDST have high accuracy in differential diagnosis of ACTH-dependent CS [8, 9, 27]. As a whole, a positive response was observed in 89.4% and 91.4% of patients with CD, and in 3.8% and 23.1% of patients with EAS, respectively. More importantly, the combination of concordant positive responses to CRH test and HDDST reaches 100% specificity and PPV, thus allowing the diagnosis of CD irrespective of MRI findings. Otherwise, a single-test approach is not able to reach a specificity of 100%. The same performance is maintained in the subgroup of patients with negative MRI or with a microadenoma < 6 mm. Furthermore, in this subgroup, a negative response to both CRH test and HDDST is sufficient to make the diagnosis of EAS.

Interestingly, in CD patients, the response rate to CRH test, as far as ACTH and cortisol percentage increase, were significantly higher in patients with microadenomas or negative imaging in respect to those with macroadenomas. A similar observation was recently reported in a group of 149 CD patients where macroadenomas tended to show a lower increase of ACTH after CRH compared to microadenomas [9]. As a negative correlation between baseline secretion and ACTH and cortisol responses to CRH in CD patients has been described [31], suggesting in this context a different degree of negative feedback impairment at the pituitary level, the finding of higher baseline ACTH levels in our patients may represent the most likely explanation for this observation.

Accordingly, the highest rate of false negative responses to dynamic tests were observed in patients with macroadenomas, in which a false negative result to both CRH and HDDST was recorded in four cases; nevertheless, in this condition BIPSS is already overlooked due to the low pretest probability of the co-existence of a pituitary macroadenoma and an ectopic CS.

The role of DDAVP test in differential diagnosis of ACTH-dependent CS is still controversial and a high frequency of false positive results in patients with EAS has been reported [2]. However, in a recent work including 167 patients with CD and 27 patients with EAS, the positive response to both CRH and DDAVP test showed a positive predictive value of 100% for CD in patients with negative MRI and negative computed tomography scan [32]. In our study, similarly to CRH test and HDDST, also the combination of positive responses to both CRH and DDAVP tests reaches a specificity and PPV of 100% for the diagnosis of CD. However, DDAVP test presents low sensitivity and specificity, thus resulting in a high prevalence of false negative and false positive results as well as a concordance rate significantly lower than that observed for CRH test and HDDST in patients with negative MRI or with a microadenoma < 6 mm. In addition, in four of these patients we recorded a concordant negative response to CRH and DDAVP tests that might have resulted in misdiagnosis. Therefore, our data indicate that DDAVP test may represent a valid alternative, in particular when discordant results arise from other dynamic tests, but CRH test, HDDST and their combination perform better and reduce the need to perform BIPSS.

On the other hand, it is well recognized that DDAVP may have an important role in the post-surgical follow-up of CD patients, as the persistence or reappearance of a positive response may precede the clinical recurrence of disease [21, 22, 33–38].

In our series, BIPSS confirmed the diagnosis of CD in 28 out of 30 patients who underwent this procedure. Two negative cases included one patient with a pituitary adenoma sized between 6 and 10 mm but discordant CRH test and HDDST and another one with negative imaging and concordant tests. Notably, in the latter case, a borderline central/periphery ratio of 2.91 was recorded. Nevertheless, diagnosis of CD was subsequently proven by remission after neurosurgery, suggesting that BIPSS returned a false negative result in both patients. The proportion of false negative we observed is in line with previous literature data reporting a prevalence of 3–19%, possibly related to anatomical or biochemical variations of disease [14, 17, 27, 30, 39, 40]. Furthermore, BIPSS is burdened by possible complications. In particular, minor adverse events (i.e., groin hematoma, tinnitus, otalgia) have been reported in about 4% of patients, while severe complications (i.e., brainstem infarction, subarachnoid haemorrhage, pulmonary and deep venous thrombosis) are expected in less than 1% of cases [27, 30]. As reported above, in our series one patient died 24 h after BIPSS due to cardiac rupture, while no complications in the other subjects were recorded. Although our fatal event was unlikely related to the procedure and complications are rare, all these observations point out the need for an accurate selection of patients referred to BIPSS.

Following the results of diagnostic performance analysis, in those patients with concordant positive responses to CRH test and HDDST but inconclusive neuroradiological findings (i.e., negative imaging or pituitary adenoma < 6 mm), the execution of BIPSS did not improve surgical outcome. Then, our data do not support the routine use of BIPSS in this subgroup of CD patients, in whom BIPSS could have been avoided in 22 out of 29 subjects. In this setting, contrarily to what the current guidelines propose [7, 13, 18, 19], CRH test and HDDST seems to be sufficient to confirm the diagnosis of CD and to provide indication to pituitary surgery. Similarly, a negative response to both tests pointed toward EAS diagnosis; in this circumstance BIPSS can be avoided too. Indeed, the present study does not propose to remove BIPSS from the diagnostic work-up of ACTH-dependent CS diagnosis, but to restrict its use when really necessary.

Our study has some limitations: first, its retrospective nature, leading in particular to an inhomogeneous selection of patients referred to BIPSS. Second, our data do not allow to draw conclusions about patients with intermediate pituitary lesion between 6 and 10 mm. Although our approach was to avoid BIPSS even in case of discordant results, except in the presence of clinical features suggestive for ectopic CS (rapid onset, hypokalemia, advanced age), these cases can still represent matter of debate.

On the other side, the strength is represented by the comprehensive and punctual biochemical and diagnostic characterization of patients which in our view makes our results very reliable.

In conclusion, our study confirms that CRH test, DDAVP test and HDDST have high accuracy in the differential diagnosis of ACTH-dependent CS. In particular, the combination of CRH test and HDDST allows to achieve the best performance in terms of sensitivity and specificity. In patients with negative MRI or with a microadenoma < 6 mm, the presence of concordant positive response to CRH test and HDDST or to CRH test and DDAVP test seems to be sufficient to establish the diagnosis of CD. In this subgroup of patients, BIPSS should be therefore reserved for those cases with discordant tests.
